# A highly divergent South African geminivirus species illuminates the ancient evolutionary history of this family

**DOI:** 10.1186/1743-422X-6-36

**Published:** 2009-03-25

**Authors:** Arvind Varsani, Dionne N Shepherd, Kyle Dent, Aderito L Monjane, Edward P Rybicki, Darren P Martin

**Affiliations:** 1 School of Biological Sciences, University of Canterbury, Private Bag 4800, Christchurch, New Zealand; 2 Electron Microscope Unit, University of Cape Town, Rondebosch, Cape Town, 7701, South Africa; 3 Department of Molecular and Cell Biology, University of Cape Town, Rondebosch, Cape Town, 7701, South Africa; 4 Institute of Infectious Disease and Molecular Medicine, University of Cape Town, Observatory, Cape Town, 7925, South Africa

## Abstract

**Background:**

We have characterised a new highly divergent geminivirus species, Eragrostis curvula streak virus (ECSV), found infecting a hardy perennial South African wild grass. ECSV represents a new genus-level geminivirus lineage, and has a mixture of features normally associated with other specific geminivirus genera.

**Results:**

Whereas the ECSV genome is predicted to express a replication associated protein (Rep) from an unspliced complementary strand transcript that is most similar to those of begomoviruses, curtoviruses and topocuviruses, its Rep also contains what is apparently a canonical retinoblastoma related protein interaction motif such as that found in mastreviruses. Similarly, while ECSV has the same unusual TAAGATTCC virion strand replication origin nonanucleotide found in another recently described divergent geminivirus, Beet curly top Iran virus (BCTIV), the rest of the transcription and replication origin is structurally more similar to those found in begomoviruses and curtoviruses than it is to those found in BCTIV and mastreviruses. ECSV also has what might be a homologue of the begomovirus transcription activator protein gene found in begomoviruses, a mastrevirus-like coat protein gene and two intergenic regions.

**Conclusion:**

Although it superficially resembles a chimaera of geminiviruses from different genera, the ECSV genome is not obviously recombinant, implying that the features it shares with other geminiviruses are those that were probably present within the last common ancestor of these viruses. In addition to inferring how the ancestral geminivirus genome may have looked, we use the discovery of ECSV to refine various hypotheses regarding the recombinant origins of the major geminivirus lineages.

## Background

The geminiviruses (Family *Geminiviridae*) are a diverse group of viruses with circular single stranded DNA (ssDNA) genomes that are composed of one or two components of 2700–3000 bp, characteristically encapsidated within twinned incomplete icosahedral (or geminate) particles. They are responsible for various economically significant crop diseases throughout the tropical and sub-tropical regions of the world [1] but are a particularly serious problem in Africa, where they threaten production of the continent's two main food crops, maize and cassava [2].

**Figure 1:**
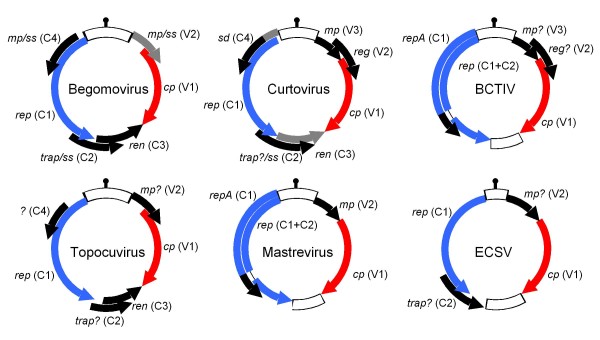
**The arrangement of genes and open reading frames (ORFs) within various major geminivirus lineages**. BCTIV = beet curley top Iran virus. ECSV = Eragrostis curvula streak virus (reported for the first time in this paper). In the case of begomoviruses only the DNA-A/DNA-A-like genome component sequence is represented. Arrows indicate the positions and orientations of numbered ORFs (V = virion sense and C = complementary sense) that are known or strongly suspected to encode expressed proteins. *mp *= movement protein gene [4-7], *cp *= coat protein gene, *rep *= replication associated protein gene, *ren *= replication enhancer gene, *trap *= transcription activator protein gene, *ss *= silencing suppressor encoding ORF [8-14], *sd *= symptom determinant encoding ORF [15,16]; *reg *= potentially encoding a protein that regulates relative ssDNA and dsDNA concentrations [4]. A question mark indicates that an ORFs function is either completely unknown or only suspected. The only genes shared between all genomes are *rep *(in blue) and *cp *(in red). Variations in the presence or size of ORFs between members of the different geminivirus groups are indicated in grey. Intergenic regions are represented as open blocks and the probable hairpin structure at the origin of virion strand replication is indicated at the 12 o'clock position.

Based on host ranges, vector specificities, genome organizations and genome-wide sequence similarities, the family *Geminiviridae *is split into the *Begomovirus*, *Curtovirus, Topocuvirus *and *Mastrevirus *genera. The mastreviruses are both the most divergent and the most distinctive of the four divisions: whereas the begomoviruses, curtoviruses and topocuviruses share superficially similar genome structures (Figure 1) and are only known to naturally infect dicotyledonous plants, mastreviruses have unique genomic features and have been found infecting both monocotyledonous and dicotyledonous plants [3].

While other currently described genomes from generic begomo-, curto- and topocuviruses contain between five and eight genes and have one intergenic region (IR), mastrevirus genomes contain only three genes and have both a large (LIR) and a small (SIR) intergenic region. Of the coding regions only the coat protein (*cp*) and replication associate protein (*rep*) genes are obviously conserved amongst all geminiviruses (Figure 1). Whereas probable movement protein (*mp*) genes occur in similar genomic locations in most geminvirus genomes there is no detectable sequence similarity in these genes between the viruses in different genera. Similarly, positional homologues of transcription activator (*trap *or *trap*-like), replication enhancer (*ren*) and symptom determinant or silencing suppressor (C4) genes which share either undetectable or only very low degrees of sequence similarity across different genera are only found in the begomoviruses, topocuviruses and curtoviruses.

Besides obvious differences in gene content there are also many subtle biologically relevant architectural differences between the genomes of mastreviruses and other geminiviruses. Prime among these are the occurrence in mastreviruses of (1) alternatively spliced introns within their *mp *[17] and *rep *genes [18], (2) a RepA protein isoform of Rep [19], (3) a canonical retinoblastoma related protein interaction motif within Rep and RepA proteins [20-22], and (4) a unique arrangement of probable Rep binding sites surrounding the virion strand origin of replication (v-*ori*) [23-26].

The evolutionary relationships between the different geminivirus genera are difficult to disentangle due in large part to the fact that genetic recombination has probably featured prominently in the evolution of these genera. It is clear, for example, that begomovirus, topocuvirus and curtovirus *rep *sequences share a far more recent common ancestor than their *cp *sequences. As the *cp *sequences of topocuviruses and curtoviruses share slightly higher degrees of sequence similarity with mastreviruses than with begomoviruses this has been interpreted as indicating that the topocuvirus and curtovirus genera may have arisen through separate recombination events between ancestral begomovirus and mastrevirus lineages [27-29]. That such recombination events may have occurred is plausible in light of the fact that there is good evidence of ongoing recombination between the *rep *sequences of curtoviruses, begomoviruses and topocuviruses [30-32]. It has been determined, for example, that since the divergence of the Old and New World begomoviruses there have been at least five separate inter-genus recombination events between curtoviruses and begomoviruses in which viruses in both genera have served as either donors or recipients of *rep *sequences [30,31]. It should, however, be pointed out that it is often very difficult to determine the polarity of sequence exchanges even amongst such well sampled virus lineages as the begomoviruses. Despite the strong possibility that one or more of the geminivirus genera may have arisen through an inter-genus recombination event, with only four major geminivirus lineages having been sampled it is impossible to definitively identify which genera are recombinant and which are parental.

Given that the root of the geminivirus evolutionary tree is unknown, and will possibly always remain so, it is also impossible to determine which geminivirus genera share more recent common ancestry. It is, for example, incorrect to assume either that the midpoint between the two most dissimilar sequences in a phylogenetic tree is the root of the tree or that the relative degrees of sequence identity shared between pairs of sequences is perfectly correlated with their evolutionary relatedness. Put another way, it often happens that two sequences which are more similar to one another than either is to a third sequence actually share a more distant common ancestor than the third sequence shares with one of the two. In the case of the geminivirus recombination debate the slightly higher degrees of similarity shared in the CP by the topocuviruses and curtoviruses with the mastreviruses has prompted the possibly incorrect assertion that topocuvirus and curtovirus CP sequences share a more recent common ancestor with the mastreviruses than they do with the begomoviruses [27,29].

Even if the begomoviruses, curtoviruses and topocuviruses do share a more recent *rep *common ancestor and the mastreviruses, curtoviruses and topocuviruses share a more recent *cp *common ancestor, this does not necessarily imply that topocuviruses and curtoviruses are the recombinant offspring of mastreviruses and begomoviruses. It is, for example, possible that the ancestral mastrevirus obtained a divergent *rep *from a geminivirus lineage that has remained unsampled. Similarly, transfer of a divergent *cp *from such a lineage to the ancestral begomovirus might explain the apparent uniqueness of this gene in begomoviruses.

The importance of considering these alternative hypotheses has been emphasized by the recent discovery of an unusual geminivirus infecting sugar beet plants in Iran: Beet curly top Iran virus (BCTIV) [33]. Whereas this virus expresses an obviously curtovirus-like CP, it expresses a mostly mastrevirus-like Rep. Although the Rep of BCTIV is only slightly more similar to those of mastreviruses than it is to those of curtoviruses, begomoviruses and topocuviruses, the *rep *gene has a distinctly mastrevirus-like structure including a probable (although currently unproven) intron. Unsurprisingly, this sequence was identified by its discoverers as a mastrevirus-curtovirus recombinant. While it represents the best indirect evidence yet that major geminivirus lineages may have arisen through inter-genus recombination, it should again be stressed that it cannot be definitively determined whether it is the mastreviruses, curtoviruses or the unique lineage represented by this new geminivirus species that is recombinant. In fact, a relatively plausible argument is that this new lineage represents none other than the mastrevirus-like virus that is speculated to have recombined with a begomovirus to yield the curtoviruses [28,29]. Differentiating between the many possible recombination hypotheses will clearly require the discovery and characterization of additional divergent geminivirus lineages.

Here we describe another highly divergent geminivirus lineage that shares traits with both begomoviruses, curtoviruses and topocuviruses on the one hand and mastreviruses on the other. This new virus is a virtual mirror image of BCTIV in that it was isolated from a monocotyledonous host, has a clearly begomovirus/curtovirus/topocuvirus-like *rep *and a mastrevirus-like *cp*. Besides emphasizing the probable importance of host-range switching and recombination in the early evolution of geminiviruses, this new geminivirus sheds further light on the possible genome arrangement of the last common geminivirus ancestor.

## Results and discussion

### Discovery of a highly divergent geminivirus lineage

Six *Eragrostis curvula *plants presenting with streak symptoms similar to those encountered in maize streak virus (MSV) infected grasses [see Additional file 1] were sampled within 40 km of one another in the KwaZulu Natal province of South Africa between December 2007 and May 2008. *Eragrostis curvula *is a perennial grass with a distribution from southern Africa to East Africa. Following *Phi*29 polymerase amplification of total extracted DNA, ~2.7 Kb *Bam*HI and *Pst*I fragments were cloned and sequenced to reveal what appeared to be a set of closely related (>95% identical) geminivirus-like genomes [GenBank: FJ665629 – FJ665634]. BLASTx (translated query nucleotide scanned against translated nucleotide sequences in the NCBI non-reduntant nucleotide sequence database) searches using the full genome nucleotide sequences of these isolates indicated significant (E score < 10^-4^), albeit low, identity matches to both mastrevirus *cp *[best match = Wheat dwarf virus (WDV)] and begomovirus *rep *[best match = Corchorus golden mosaic virus (CoGMV)] translated amino acid sequences. Electron microscopic analysis of negatively stained leaf sap from a symptomatic *E. curvula *plant (isolate ECSV [Gre5_Ky6-2008]) indicated the presence of geminate particles [see Additional file 1] and supported our conclusion that the new virus was most likely a highly divergent monocot infecting geminivirus lineage – hereafter referred to as Eragrostis curvula streak virus or ECSV.

**Figure 2:**
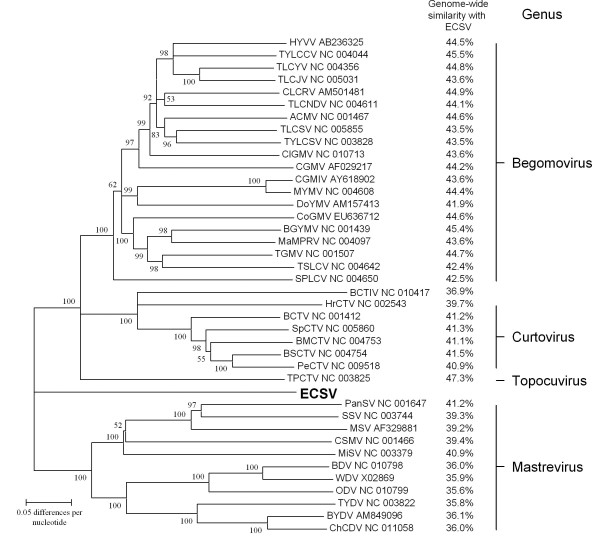
**Degrees of genome-wide sequence identity shared between ECSV (in bold; isolate ECSV [Za-Gre3-g257-2007]) and 40 representative geminivirus genomes (or DNA-A or DNA-A like genome components in the case of the begomoviruses; virus names and GenBank accession numbers are given in the tree)**. Note that due to (i) recombination during the evolutionary histories of many of the represented viruses (ii) very high degrees of alignment uncertainty and (iii) the strong possibility that many regions of the aligned genomes are not homologous, the presented neighbour joining tree is simply intended as a graphical depiction of genome-wide nucleotide sequence identities rather than a credible representation of the evolutionary relatedness of these viruses. Numbers associated with tree branches indicate percentage of bootstrap support (from 1000 replicates) for those branches. Branches with less than either 50% bootstrap support or less than 90% interior branch length test support (as determined in MEGA 4.0) have been collapsed. The percentage genome-wide nucleotide sequence identities shared between ECSV and the other geminivirus genomes (with alignment gaps treated as missing data rather than a fifth character state) is presented on the right.

Attempts to align one of the ECSV genome sequences to those of 40 representative geminiviruses proved largely futile due to the large genetic distances separating ECSV from other currently described geminiviruses. Nevertheless, a neighbor joining phylogenetic tree constructed from this alignment using genetic distances calculated without any evolutionary model (called p-distances in MEGA) serves as a reasonable graphical depiction of the degrees of genome-wide sequence identity shared between the ECSV genome and those of other geminiviruses (Figure 2).

### The ECSV genome displays a mixture of mastrevirus- and begomovirus/topocuvirus/curtovius-like characteristics

The arrangement of open reading frames (ORFs) within the ECSV genome is similar to those described previously for other geminiviruses (Figure 1; [see Additional file 2]): The locations of the two virion sense ORFS (V1 and V2) respectively correspond with the positions of *cp *and *mp *and genes found in other geminivirus genera. Similarly, the two complementary sense ORFs (C1 and C2) occur in the same positions as *rep *and *trap*/*trap*-like genes found in topocuviruses, begomoviruses and curtoviruses. Whereas the predicted expression products of the V1 and C1 ORFs share easily identifiable similarities with the geminivirus CP and Rep proteins, respectively, the other ECSV ORFs had no obvious homologues amongst sequences currently deposited in public databases. For example, whereas the V2 ORF is in an analogous position to the *mp *genes of other geminiviruses, its translated sequence lacks the large hydrophobic domain that characterizes mastrevirus MPs [17] and shares no significant amino acid sequence similarity with any described proteins of geminiviruses (BLAST E> 0.19), ssDNA viruses (BLAST E> 0.47), viruses in general (BLAST E> 0.43), or any other organisms (BLAST E> 6.1).

The C2 ORF in the ECSV genome is a positional analogue of the begomovirus, topocuvirus and curtovirus *trap/trap*-like genes and, as with these genes in curtoviruses and begomoviruses, it partially overlaps the Rep C-terminus encoding part of *rep*. Although we found marginal evidence that the C2 ORF of the new virus is a genuine homologue of *trap *(E-score = 0.083 when restricting BLASTp comparisons to geminivirus proteins) it is important to point out that the region of the potential C2 expression product contributing to this significant similarity is that encoded by the portion of C2 that overlaps *rep*. This marginal similarity might therefore simply reflect *rep *conservation rather than any significant degree of sequence similarity or functional conservation between the C2 ORF of the new virus and the *trap *genes of other geminiviruses. It is noteworthy, however, that we were able to identify potential transcription factor binding sites 72 nucleotides upstream of the C2 start codon [see Additional file 2] that are nearly identical to those identifiable in curtoviruses, begomoviruses and topocuviruses and which have been shown to strongly influence *trap *expression in begomoviruses [34].

As with mastreviruses and the newly characterized BCTIV sequence, the new genome contains two probable intergenic regions. However, in the case of ECSV, the analogue of the mastrevirus LIR and the begomovirus, curtovirus and topocuvirus IR (i.e. the presumed location of both the *v-ori *and transcription start sites) is apparently smaller than the analogue of the mastrevirus SIR (i.e. the presumed location of both the complementary strand replication origin and transcription termination sites). To avoid confusion we refer to the v-*ori *containing IR as IR-1 and the larger IR as IR-2.

We identified a number of sequence elements within IR-1 and IR-2 that, by analogy with other geminiviruses, are potentially involved in replication and/or transcription [see Additional file 2]. The most interesting among these was the presumed nonanucleotide sequence at the v-*ori *that falls within the loop sequence of a probable IR-1 hairpin structure. As with BCTIV [33], ECSV has a TAAGATTCC sequence rather than the usual TAATATTAC sequence found in almost all other geminiviruses.

Besides this similarity with BCTIV, the overall structural arrangement of IR-1 is most similar to the IRs of begomoviruses, curtoviruses and topocuviruses. Directly repeated sequences in IR-1 between the probable *rep *initiation codon and a potential complementary-sense transcript TATA box, resemble the arrangement of begomovirus, topocuvirus and curtovirus iterated sequence elements implicated in v-*ori *recognition and binding by Rep [23,24,36].

Also unlike BCTIV and the mastreviruses, the *rep *gene of ECSV is probably translated from an unspliced complementary strand transcript. The predicted protein encoded by this gene contains various rolling circle replication and deoxyribonucleotide triphosphate binding motifs that characterize all known geminivirus Rep proteins. Interestingly, it also contains a canonical LXCXE retinoblastoma binding (Rb) protein interaction motif which, unlike analogous Rb interaction domains identified in other geminiviruses, is close to the C-terminus of the protein [see Additional file 3].

### The evolutionary relationships amongst major geminivirus lineages

**Figure 3:**
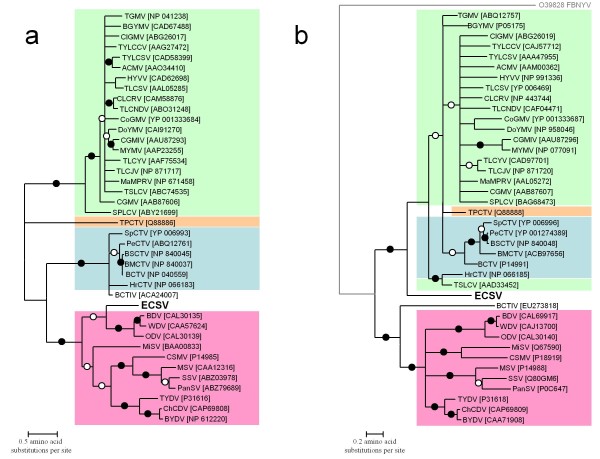
**Maximum likelihood trees of (a) coat protein (JTT + G_4 _model) and (b) replication associated protein (RtRev + G_4 _model) amino acid sequences of ECSV (isolate ECSV [Za-Gre3-g257-2007]) and 40 other viruses representing the broadest breadth of currently sampled geminivirus diversity**. Whereas the CP tree is unrooted, the Rep tree was rooted using the translated "master" *rep *sequence of Faba bean necrotic yellows virus (FBNYV; in grey). Viruses that are clearly members of the currently established geminivirus genera, *Begomovirus*, *Topocuvirus*, *Curtovirus *and *Mastrevirus *are indicated in green, orange, blue and pink respectively. Branches of the tree marked with filled circles were present in 90 or more maximum likelihood tree bootstrap replicates (performed in PHYML)and more than 99% of constructed trees from alignments sampled during the statistical alignment process (performed in STATALIGN). Open circles represent branches supported by 70 or more percent of the maximum likelihood tree bootstrap replicates and 95 or more percent of trees constructed during statistical alignment. Branches were collapsed if they were not supported in the consensus trees of either the maximum likelihood bootstrap replicates or the statistical alignment process. Branches were also collapsed if they were supported in less than either 50% of the bootstrap replicates or 90% of the trees generated during the statistical alignment.

As *cp *and *rep *were the only ECSV genes that were obviously homologous to those of other geminiviruses we focused on these to explore the possible evolutionary relationships between ECSV and the other geminiviruses. We constructed phylogenetic trees for CP and Rep from translated amino acid sequences using two separate approaches. In the first we aligned the amino acid sequences using CLUSTALW, used PROTTEST to determine the best fit models of amino acid substitution, and constructed bootstrapped maximum likelihood phylogenetic trees using PHYML. As there is a large degree of uncertainty associated with aligning such divergent amino acid sequences, we also used the program STATALIGN to directly construct phylogenetic trees in which alignment uncertainty is explicitly accounted for. We then used the absolute consensus of the PHYML and STATALIGN trees, collapsing all tree branches that were: (i) Not retrieved in the consensus trees generated by both methods; (ii) were supported in less than 50% of the PHYML bootstrap replicates; or (iii) were only represented in less than 90% of the trees constructed from sampled alignments during the statistical alignment process (Figure 3).

While the predicted CP amino acid sequence of ECSV is clearly most similar to those of the Eurasian mastreviruses WDV, Barley dwarf virus (BDV) and Oat dwarf virus (ODV; Figure 3a), its Rep amino acid sequence is apparently most closely related to those of begomoviruses, curtoviruses and topocuviruses (Figure 3b).

Note, however, that whereas the Rep phylogeny could be rooted using a nanovirus Rep sequence as an outlier, this was not possible for the CP phylogeny since there are no obvious homologues of geminivirus CPs in any other virus group. Therefore, although we may have some confidence that the Rep phylogeny represents the flow of time from left to right, the same is not true for the CP phylogeny. It is possible, for example, that the root of the CP tree is located on the branch separating the ECSV lineage from that of WDV, BDV and ODV. If the root of the CP tree were on this branch then time would run from right to left into the tree through nodes separating ECSV from the begomoviruses and topocuviruses and from left to right through all other nodes.

Therefore, while the apparent discrepancy between the location of ECSV sequences in the Rep and CP phylogenies might indicate that ECSV is, along with the topocuviruses, curtoviruses and BCTIV, yet another example of an inter-genus geminivirus recombinant, it is possible that the apparent discrepancy is an artifact of incorrect rooting.

### Analysis of inter-genus recombination in geminiviruses

Directly testing for recombination in potential inter-genus geminivirus recombinants is not straightforward in that it requires the accurate alignment of extremely diverged nucleotide sequences. Inter-genus recombination has, however, been quite easily detected in curtoviruses, topocuviruses and begomoviruses where the recombination events in question (involving *rep *sequence exchanges) have occurred in the relatively recent past. For these recombination events nucleotide sequence similarities in different parts of recombinant genomes are closely related to different parents, despite the parental sequences being very different from one another [30-32,36,27,29].

To test for recombination, we first constructed a nucleotide sequence alignment with ECSV, BCTIV and one representative from each of the four established geminivirus genera and tested this for recombination using an approach which rigorously accounts for the adverse influences that alignment inaccuracies have on recombination analysis [37].

**Figure 4:**
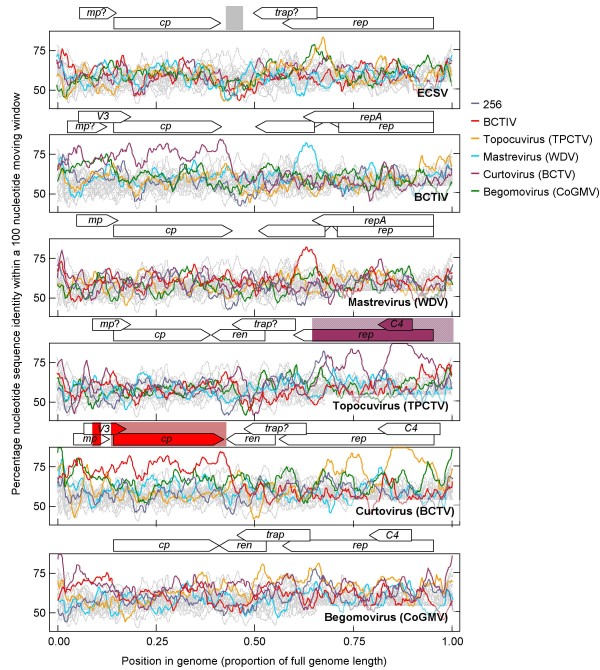
**Pairwise genome scans of local nucleotide sequence similarities (uncorrected by any evolutionary model – corresponding to p-distances in MEGA 4.0 with pairwise deletion of gaps) within a moving 100 nucleotide window between ECSV (isolate ECSV [Za-Gre3-g257-2007]), BCTIV and representatives of the four established geminivirus genera**. Each coloured plot represents a different pairwise nucleotide sequence alignment (using CLUSTALW with a gap open penalty of 6 and gap extension penalty of 3) between a single representative of each of the six main geminivirus lineages and representatives of all the other lineages. The grey plots represent analogous scans between 20 geminivirus genome pairs in which the positions of nucleotides have been randomly reshuffled and aligned using the same settings used to align the unshuffled nucleotide sequences. The maximum and minimum bounds of these scans represent the degrees of sequence similarity expected following alignment amongst unrelated sequences with the same nucleotide composition as the real geminivirus sequences.

While our analysis (Figure 4) supported the prevailing hypotheses that the curtoviruses and topocuviruses are inter-genus recombinants [27-29], it also indicated that BCTIV is probably not an inter-genus recombinant as suggested by Yazdi *et al*. [33]. BCTIV is instead identified as a close relative of the "mastrevirus-like" progenitor formerly proposed by Stanley *et al*. [28] and Rybicki [29] as the originator of the curtovirus coat protein gene. While our analysis also indicated that topocuviruses are the recombinant offspring of begomoviruses and curtoviruses, the identified recombination event in *rep *is within an extremely recombinogenic genome region such that it is very probable that either one or both of the identified parental sequences (i.e. the begomovirus CoGMV and the curtovirus Beet curly top virus [BCTV]) are also inter-species recombinants in this genome region [30-32]. The possibility of quite widespread ongoing *rep *sequence recombination amongst the begomoviruses, topocuviruses and begomoviruses is, for example, strongly supported by the fact that these lineages cannot be reliably resolved within the Rep amino acid sequence phylogeny (Figure 3b).

Importantly, our direct recombination and nucleotide alignment tests indicated that the the *cp *and *rep *genes of ECSV are not detectably derived by recombination from mastrevirus and curtovirus/begomovirus/topocuvirus ancestors. Despite the apparent similarity in CP amino acid sequence shared by ECSV and the Eurasian wheat, barley and oat dwarf mastreviruses (Figure 3a), the ECSV *cp *nucleotide sequence cannot, for the most part, be meaningfully aligned with that of WDV (in the top panel of Figure 4 note how the multiple grey lines of the alignment controls overlap the blue line representing the ECSV-WDV alignment).

The only evidence that we could find for a recombinant origin of ECSV was that the genome region corresponding to IR-2 is highly divergent relative to the analogous genome region in other geminiviruses, and has potentially been derived through recombination from either a highly divergent geminivirus lineage or another source entirely. Given the extremely distant relationship between ECSV and the other geminiviruses and the fact that this recombination hypothesis invokes the existence of a still more divergent geminivirus lineage, it is very difficult to discount the alternative hypothesis that this recombination signal is simply an alignment artifact.

## Conclusion

ECSV represents a new genus-level geminivirus group displaying a mixture of characteristics normally associated with specific geminivirus genera. Accordingly, we propose the name Ecuvirus for a new genus of *Geminiviridae*. As the most divergent geminivirus species yet identified, the genome features that ECSV shares with other geminiviruses provides some indication of what the last common ancestor of the geminiviruses may have looked like. It is, for example, probable that this ancient progenitor expressed a mostly topocuvirus, curtovirus and begomovirus-like Rep protein from an intronless transcript that, unlike the Reps of these other viruses, contained a canonical LXCXE pRBR interaction motif. While this virus may have had movement and transactivation protein-like genes it probably had neither a C4 gene nor a replication enhancer protein gene. It also probably had a virion sense replication origin structure more closely resembling that of the begomoviruses, curtoviruses and topocuviruses than that of the mastreviruses. The lack of a replication enhancer gene probably meant that, like the mastreviruses, this earliest geminivirus had two intergenic regions.

Besides indicating how ancient geminiviruses may have looked, viruses such as ECSV may also provide some clues as to their biology. The fact that ECSV was found infecting a monocotyledonous host does not necessarily imply that the earliest geminiviruses infected monocots but it does indicate that major host-range switches between dicotolydonous and monocotyledonous plants have occurred multiple times during geminivirus evolution. Therefore, unless many more extremely divergent geminivirus lineages are discovered, it could prove extremely difficult to determine whether monocots or dicots were the first geminivirus hosts. It may in fact be considerably easier to identify the earliest geminivirus vectors. Although the ECSV CP is clearly very similar to those of mastreviruses, it is not obviously derived from mastreviruses through recombination. The possibility therefore remains that the real root of the geminivirus CP tree is somewhere along one of the mastrevirus or ECSV associated branches – a possibility that, if confirmed, would indicate not only that the last common ancestor of all geminiviruses had a very mastrevirus/ECSV-like coat protein, but that it was also probably leafhopper transmitted.

## Methods

### Virus Sampling

Six grasses presenting with mild maize streak virus-like symptoms [see Additional file 1] were sampled in KwaZulu Natal province of South Africa between December 2007 and May 2008 [see Additional file 4 for sampling coordinates]. The host species of the infected grasses was identified as *Eragrostis curvula *(common name, weeping love grass) based on sequencing of chloroplast *ndhF *genes as described by Varsani *et al*. [38].

### Electron Microscopy

Approximately 10 μl of leaf sap was obtained from fresh leaf material (sample ECSV [Gre5_Ky6-2008]) and diluted with 50 μl 0.1 M sodium acetate buffer (pH 4.8). 10 μl of the diluted sap was negatively stained with 2% uranyl acetate on carbon-coated copper grids and observed in a JEOL 1200CX transmission electron microscope.

### Genome cloning and sequencing

Viral genomes were isolated from infected grass samples [39] and amplified using *Phi*29 DNA polymerase (TempliPhi™, GE Healthcare, USA) as described previously [40,41]. Amplified full-genome concatemers were digested with either *Bam*HI or *Pst*I to yield ~2.7-kb linearised viral genomes which were inserted into pGEM 3 Zf+ (Promega Biotech) cloning vector. Both strands were sequenced using primer walking by Macrogen Inc. (Korea). Sequences were assembled and edited using DNAMAN (version 5.2.9; Lynnon Biosoft) and MEGA version 4 [42].

### Identification of genes

We identified all open reading frames (ORFs) that could potentially express proteins larger than 50 amino acids in length and used protein-protein BLAST (BLASTp) [43] searches to identify potential homologues of these in the NCBI non-redundant protein sequences database. To increase our chances of finding significantly similar protein sequences in this database, we used a nested search strategy initially restricting the search to geminivirus protein sequences, and then progressively expanding the search to include all ssDNA virus protein sequences, all virus protein sequences and, finally, all publically available protein sequences irrespective of their origin. For ORFs where no matches were found we additionally searched the NCBI environmental sample, whole genome shotgun read, high throughput genomic sequence and expressed sequence tag databases using tBLASTn. Identified proteins with BLAST E scores smaller than 0.1 were considered to potentially have a common ancestry with our query sequences.

### Phylogenetic and recombination analysis

Translated amino acid sequences of *cp *and *rep *gene sequences from 39 representative geminivirus species were obtained from public databases and aligned using the CLUSTALW (gap open = 2, gap extension = 0.1) [44] implementation in MEGA. Maximum likelihood phylogenetic trees were constructed using PHYML[45] with model parameters selected using either PROTTEST (for amino acid sequence alignments) [46] or the automated model selection procedure implemented in RDP3.32 (for full genome nucleotide sequence alignments) [47]. To account for uncertainty in the amino acid sequence alignments used to reconstruct phylogenetic relationships, alignment and phylogenetic tree construction were simultaneously carried out using STATALIGN[48] using 10^6 ^MCMC updates with the first 10^5 ^updates discarded as burn-in (as judged by the convergence statistic provided by STATALIGN) and the same basic evolutionary model (but without rate variation) suggested for each gene/genome sequence alignment by PROTTEST. The consensus of 1000 trees constructed from alignments sampled during the statistical alignment process was retrieved using the CONSENSE component of PHYLIP[49].

Nucleotide/amino acid sequence similarities (using p-distances and pairwise deletion of gaps) were calculated using MEGA. The degrees of similarity shared by full-length genome sequences of various representative geminiviruses (see Figure 2) were graphically depicted using a neighbor joining tree (1000 bootstrap replicates, p-distances) constructed in MEGA. Pairwise nucleotide sequence similarity plots for individual representatives of six major geminivirus lineages were plotted using optimal pairwise CLUSTALW nucleotide sequence alignments (gap open = 6; gap extension = 3) using RDP3.32 with a window size = 100 nucleotides and a step size = 5 nucleotides.

Recombination was analysed using the RDP[50], GENECONV[32], BOOTSCAN[51], MAXCHI[52], CHIMAERA[53], SISCAN[54] and 3SEQ[55] methods implemented in RDP3.32. The method developed by Varsani *et al*., [37] for detecting recombination in difficult to align sequences was followed. Briefly, this involved a three stage recombination signal verification process in which (i) a recombination signal detected in a particular triplet of sequences using a nucleotide sequence alignment generated using the CLUSTALW method was retested with: (ii) the same recombination analysis methods following realignment with the POA method [56] and (iii) using a chi square test of re-alignment consistency described previously [37].

## List of abbreviations used

BCTV: Beet curly top virus; BDV: Barley dwarf virus; BCTIV: Beet curly top Iran virus; CP: Coat protein; *cp*: Coat protein gene; CoGMV: Corchorus golden mosaic virus; ECSV: Eragrostis curvula streak virus; FBNYV: Faba bean necrotic yellows virus; IR: intergenic region; LIR: Long intergenic region; MP: movement protein; *mp*: movement protein gene; MSV: Maize streak virus; ODV: Oat dwarf virus; ORF: Open reading frame; PCR: Polymerase chain reaction; *ren*: replication enhancer protein gene; Rep: replication associated protein; *rep*: replication associate protein gene; Rb: retinoblastoma binding; *reg*: regulatory gene; *sd*: symptom determinant gene; SIR: Short intergenic region; ssDNA: Single stranded DNA; *ss*: silencing suppressor gene; TPCTV: Tomato pseudo curly top virus; *trap*: transcription activator protein gene; v-*ori: *virion strand origin of replication; WDV: Wheat dwarf virus.

## Competing interests

The authors declare that they have no competing interests.

## Authors' contributions

AV collected isolates, cloned and sequenced the viruses, analysed the data, helped prepare the manuscript and secured funding for the project's execution. DNS and AM collected isolates and helped clone and sequence viruses. KD collected isolates and conducted the electon microscopic study. EPR provided ideas and comments during manuscript preparation. DPM analysed the data and prepared the manuscript. All authors read and approved the final manuscript.

## Supplementary Material

Additional File 1 **Supplementary Figure 1.** Discovery of a divergent monocotyledonous grass infecting geminivirus. Discovery of a divergent monocotyledonous grass infecting geminivirus. (**a**) Leaves of *Eragrostis curvula *presenting with mild streak symptoms. (**b**) Negatively stained geminate particles (indicated by arrows) within the leaf sap of an MSV infected maize plant (left) and an ECSV infected *Eragrostis curvula *plant. The size bars represent 100 nm. Click here for file

Additional File 2 **Supplementary Figure 2.** Annotated ECSV genome sequence. Annotated ECSV genome sequence (isolate ECSV [Za-Gre3-g257-2007]). Sequence features that potentially play some role in ECSV replication and transcription (inferred by analogy with similar features identified in other geminiviruses) are marked in colour. [1] Stenger, *et al*., 1991. Proc Natl Acad Sci USA 88:8029; [2] Sunter, *et al*. 1985. Nucl Acids Res 13:4645; [3] Morris-Krsinich *et al*. 1984. Nucleic Acids Res 13:7237; [4] Tu & Sunter 2007. Virology 367: 117; [5] Argüello-Astorga et al. 1994. Virology 203:90. Click here for file

Additional File 3 **Supplementary Figure 3.** Annotated predicted replication-associated protein amino acid sequence of ECSV. Annotated predicted replication-associated protein amino acid sequence of ECSV (isolate ECSV [Za-Gre3-g257-2007). Potential rolling-circle replication motifs and interaction domains inferred by analogy with other geminiviruses are highlighted. [1] Argüello-Astorga et al. 2001. Arch Virol 146:1465 [2] Koonin & Ilyina. 1992. J Gen Virol, 73:2763; [3] Argüello-Astorga *et al*. 2004. J Virol 78:4817 [4] Horvath *et al*. 1998. Plant Mol Biol 38:699; [5] Orozco *et al*. 1997. J Biol Chem 272:9840. [6] Xie *et al*. 1995. EMBO J 14:4073; [7] Gorbalenya & Koonin. 1989. Nucl Acids Res 17:8413. Click here for file

Additional File 4 **Supplementary Table 1.** Geographical coordinates at which ECSV samples were collected. Click here for file
