# Supplementary figures and images for: A highly divergent South African geminivirus species illuminates the ancient evolutionary history of this family
Source: Virol J. 2009 Mar 25;6:36. doi: 10.1186/1743-422X-6-36 (PMC2666655; doi:10.1186/1743-422X-6-36)

## Slide 1
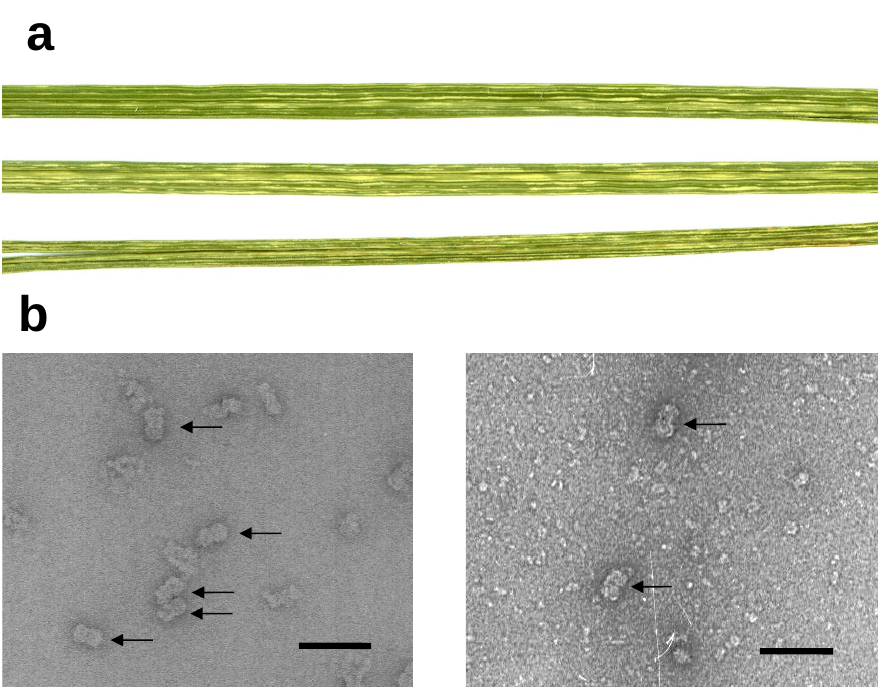

a
b

Supplement: Additional File 1 — Supplementary Figure 1. Discovery of a divergent monocotyledonous grass infecting geminivirus. Discovery of a divergent monocotyledonous grass infecting geminivirus. (a) Leaves of Eragrostis curvula presenting with mild streak symptoms. (b) Negatively stained geminate particles (indicated by arrows) within the leaf sap of an MSV infected maize plant (left) and an ECSV infected Eragrostis curvula plant. The size bars represent 100 nm. [file 1743-422X-6-36-S1.ppt]
