# Supplementary material for: A highly divergent South African geminivirus species illuminates the ancient evolutionary history of this family
Source: Virol J. 2009 Mar 25;6:36. doi: 10.1186/1743-422X-6-36 (PMC2666655; doi:10.1186/1743-422X-6-36)
Supplement: Additional File 2 — Supplementary Figure 2. Annotated ECSV genome sequence. Annotated ECSV genome sequence (isolate ECSV [Za-Gre3-g257-2007]). Sequence features that potentially play some role in ECSV replication and transcription (inferred by analogy with similar features identified in other geminiviruses) are marked in colour. [1] Stenger, et al., 1991. Proc Natl Acad Sci USA 88:8029; [2] Sunter, et al. 1985. Nucl Acids Res 13:4645; [3] Morris-Krsinich et al. 1984. Nucleic Acids Res 13:7237; [4] Tu & Sunter 2007. Virology 367: 117; [5] Argüello-Astorga et al. 1994. Virology 203:90. [file 1743-422X-6-36-S2.doc]

TAAGATTCCCGCTCCCCCCCCCCGCGAGCGCGTAGCGCGAGCCCGCCGGCCTTTTTCGAATTAAAGATGTCAGGCCCACC 80

Virion strand origin of replication [1]

Inverted repeat sequence [2]

GC-rich sequence

V2 ORF start codon

TCCCTGCGATCGCACCGAGCCAGTCCAGACAATACCTTTGTTGCTTTGCCATGGCCGGCGCAGTCTGCCGCTTTGCCATG 160

CGGAGGTCCTTCCACCACAAGACTTAGGTCAGATCCGTTACTTGGTAAAGCGATTCAAGGCCCCTGTCCTTATTAGGACG 240

Coat protein gene start codon [3]

CTTGCGGAACAGTACAGCGGCAGTTCCTTAGCAGATAAGCTTGAGCTTATCTGGACGTTCTGTGATACCTTACAACATGA 320

V2 ORF stop codon

AGCGGAAGAGGAATGAAGCAGTTCCTGCCGGTCGCCGTTATCCTCAACGGCGTCGTATGTACTACCGTCCTCGTAAGCCT 400

TTCTTCCCCCGCCCAGTGTACACAAGGTCATCCAGTGTTAAGCGTCCTGCCCTACAGATTAGTGGTCTCGTATACGGGAA 480

CTCTAGTACGGGAGCCGTTAAAATTAACACTGGAGCTCTTAGCCTTGTCACTGCATTTAAGGCGGGGACAGCTGAAGAGT 560

GCAGGCACTCTAATCAAACTATTGTTAAGTCCTTTGACATTAGTGGTACTCTATATGTTCAGTCTCCCACCAGTTCAAAC 640

TGTGGACCTGTTGTTGTTTACTTCTGGCTAATCTATGACTCAGAGCCTAGGCAGGCTATTCCTAACATAACAGATGTGTT 720

CTCCATGCCTTGGACTAGTGTGCCGTCAAGTTGGCGTATATCTCGCTCTTCTTCACACCGATTTGTTGTGAAGAGGAAGT 800

GGCATTACGAATTGATGTCAGATGGTGTCCTTCCGCAGAGTAACACGAAAGTGCAGACTCATAATCCCGTTTCTAGGAAC 880

ATGATGGACTTTTCTAAGTACATCAATAATCTTGGTGTGCCTACGGAGTGGATGAGTACTGGAGATGGAACCATTGGTGA 960

TATCAAGAAGGGCGCGTTGTACCTCGCCGCTGCCTGCCGACAGGGAATTGTTGGAGATGCAACCAAGATTACTATAGAAG 1040

Potential virion-sense gene polyadenylation signal [3]

Coat protein gene stop codon

TCGAGTTTATCGGGCAGTCTCGTACTTATTTCAAGAGCATTGGCTACCAGTGATTACTTCGCAGCACTATTGAATAAACA 1120

TGTTGAACATAATGTTGTTGTTCCTGTGAGCCGGGAGGCGCGGTCCGTCGGGGCCGAAGGCCCGACGGTTCCGCCGAGGC 1200

Potential complementary sense gene polyadenylation signals [3]

GCGAAGCGCGAAGCGCTAGCGCCGTGGAACCATATTTGCCGTTAACATTTCCTTATTTTAACGCCCCTGCGAGGCGGCGG 1280

C2 ORF stop codon

GCGAAGCCCGACGTCATGCGCGCAGCGCCTTACACAAGATTGAAACACTAACAAGAATTGATACTTTCATCCCATAGTGC 1360

CTCATTAGGGCCCATGAGGTTACAGTTACAATCCACACACTTATCTAAAATACATTGAGCGCTGTGGCGCTCCGAGAACT 1440

CAAAATAAGTGGTAGGTATGTTACATGCGTCTTCGTACTCCTGCTCAGCCCTCGACTTGAAGTGCGGCATTGGCATGAAT 1520

Rep gene stop codon

TGATGTACTCGGCCACAGTTACACGGCTTGGTCACCTGGATCACTATCTGAAGCCCACATTGGAGATTCAGGGTCATCTT 1600

CTCTGGTTTGCTCCCGCATAGTAAGTGAGACTCCGCCAAAGAGAGCATCTTGGATATCCACGAAGAGCGCATTTTTGCTA 1680

GTCCACGAACGAAGCTGGTGATTCTCTTCACAGTCAAGGAAGGATTTATAGGACTGGTCCGAGTTACAGAGCACGATAGC 1760

C2 ORF start codon

Potential C2 ORF TATA box

TGGTTTACCACCTTTAACCAGAACCGGTTTCCCATATTTCAGGTTACTTTGCCAGTCTTTCTGAGCTCCTATAAATTCCT 1840

Potential transcription factor binding sites [4]

0

TCCAATGTTTTAGATATTTAGGGTTGACGTCATCGATGACGTTGTAACTTGCCTCGTTGTCGAACACGGCTCCGTTGAGG 1920

TCCAGATGTCCGCTGAGATAGTTGTGACGTCCCAATGATCTAGCCCAAGCCGTTTTTCCTGTACGGGAATCACCTTCAAT 2000

TATCAAAGATAATGGTCTATCGGGTAAAGGATCTGCCCTTAAGTTATTTGCGACCCAGTCAGATAAGACTCTCGGTACAG 2080

AAAAACTGCTCTCTGAATACTTTGGAACAAATTCAGCTCGTACCTCAGACCATATGCGCCTGGCGTTGGTCACCAGGTTA 2160

TGGTGTTGCAGCCAGAATGTACGCGGCTCATTATCCTTCACTAGCTGCAGAGCCTCCTCAATTGAACCTGAATTCACCGC 2240

CTGATGCCACCATTCGTCCTGCTCAGCCTTCCTCTTGCGGCCCGTCAGCCTCTTGTCACATGGTACTGTACCATGTTCGT 2320

AGAAAGATCCAGCTTCTTTCTGTATGTACTTCAGGCTCTTGGATACTGAACGACAGGTCTCAATCTTCGGATGGAATTCC 2400

CCGAAGTCGAATATACGGGGGTCCCGTATATCTCTACGCTCACTTGTACATACTATAGCATGAAGATGGTTGTTACCATC 2480

TTGGTGCTTTTCCTGTTGTACGCGTACGTACACGACGTGGCTAGCAAGAGTTGAGTGGGAAGTGAGGAACTCTCCCACAT 2560

CCTTAGGCTCTCTTGGGCATTGTGAGTAAGTGAGGAAGAACGCCCTCCCTTGGATCCTAAATCTATGGCTGGATGAAGCC 2640

Rep gene start codon

Potential iteron sequences [5]

ATCCTCAAACTAGTTTGGTCAAACCAAAACCCCTGGGTCAATTGTGAGGAATGAGGATTGGGGATGTTATATAGCCTCTG 2720

Inverted repeat sequence [2]

Potential complementary- and virion-sense gene TATA box

GGTCCTCTGGGGGGGGGGGAGCGTC 2746
