# Supplementary material for: A highly divergent South African geminivirus species illuminates the ancient evolutionary history of this family
Source: Virol J. 2009 Mar 25;6:36. doi: 10.1186/1743-422X-6-36 (PMC2666655; doi:10.1186/1743-422X-6-36)
Supplement: Additional File 3 — Supplementary Figure 3. Annotated predicted replication-associated protein amino acid sequence of ECSV. Annotated predicted replication-associated protein amino acid sequence of ECSV (isolate ECSV [Za-Gre3-g257-2007). Potential rolling-circle replication motifs and interaction domains inferred by analogy with other geminiviruses are highlighted. [1] Argüello-Astorga et al. 2001. Arch Virol 146:1465 [2] Koonin & Ilyina. 1992. J Gen Virol, 73:2763; [3] Argüello-Astorga et al. 2004. J Virol 78:4817 [4] Horvath et al. 1998. Plant Mol Biol 38:699; [5] Orozco et al. 1997. J Biol Chem 272:9840. [6] Xie et al. 1995. EMBO J 14:4073; [7] Gorbalenya & Koonin. 1989. Nucl Acids Res 17:8413. [file 1743-422X-6-36-S3.doc]

Rolling circle replication motifs [2]

Potential iteron related domain [1]

MASSSHRFRIQGRAFFLTYSQCPREPKDVGEFLTSHSTLASHVVYVRVQQEKHQDGNNHLHAIVCTSERRDIRDPRIFDF 80

Position of cryptic retinoblastoma binding motif in begomoviruses [3]

Potential oligomerisation domain [4,5]

GEFHPKIETCRSVSKSLKYIQKEAGSFYEHGTVPCDKRLTGRKRKAEQDEWWHQAVNSGSIEEALQLVKDNEPRTFWLQH 160

Position of retinoblastoma binding motif in mastreviruses [6]

Mastrevirus-begomovirus consensus oligomerisation domain [4,5]

HNLVTNARRIWSEVRAEFVPKYSESSFSVPRVLSDWVANNLRADPLPDRPLSLIIEGDSRTGKTAWARSLGRHNYLSGHL 240

Potential dNTP binding motif [7]

Potential retinoblastoma binding motif

DLNGAVFDNEASYNVIDDVNPKYLKHWKEFIGAQKDWQSNLKYGKPVLVKGGKPAIVLCNSDQSYKSFLDCEENHQLRSW 320

TSKNALFVDIQDALFGGVSLTMREQTREDDPESPMWASDSDPGDQAV 367
