# Supplementary material for: A highly divergent South African geminivirus species illuminates the ancient evolutionary history of this family
Source: Virol J. 2009 Mar 25;6:36. doi: 10.1186/1743-422X-6-36 (PMC2666655; doi:10.1186/1743-422X-6-36)
Supplement: Additional File 4 — Supplementary Table 1. Geographical coordinates at which ECSV samples were collected. [file 1743-422X-6-36-S4.doc]

**Additional file 4. Geographical coordinates at which ECSV samples were collected**

| Isolate | Longitude | Latitude | Accession number |
| --- | --- | --- | --- |
| ECSV [Za-Gre4-Ky3-2008] | 30.42400 | -29.10351 | FJ665630 |
| ECSV [Za-Gre5-Ky6-2008] | 30.39723 | -29.18735 | FJ665634 |
| ECSV [Za-Esc1-g382-2008] | 30.00160 | -29.22360 | FJ665631 |
| ECSV [Za-Gre1-g261-2007] | 30.33654 | -29.17791 | FJ665633 |
| ECSV [Za-Gre2-g256-2007] | 30.42629 | -29.16067 | FJ665629 |
| ECSV [Za-Gre3-g257-2007] | 30.42629 | -29.16067 | FJ665632 |
